# Cooperation and Competition in the Innovation Ecosystem From the Perspective of Evolutionary Psychology

**DOI:** 10.3389/fpsyg.2021.769847

**Published:** 2021-11-18

**Authors:** Xiaochun Zhao

**Affiliations:** School of Management, Anhui University, Hefei, China

**Keywords:** innovation ecosystem, corporation, competition, evolutionary psychology, innovation

## Abstract

The innovation ecosystem is distinct from innovation itself; the former is based on technology, talent and markets, as well as many other elements of innovation that form the organisational system. Current research on the innovation ecosystem has primarily focused on theoretical discussions of the concept and analysis of evolution mechanisms and influential factors; however, research on mechanisms that influence cooperation and competition within the system is limited. Corporation and competition are the critical elements in the acquisition value of the innovation ecosystem and the realisation of innovation breakthroughs. Given the dynamic nature and complexity of the innovation ecosystem, this research is based on the “value co-creation” theory of innovation ecosystems and consistency with the “reciprocity” principle of evolutionary psychology. Understanding the mechanisms that underlie corporation and competition in the innovation ecosystem from the perspective of evolutionary psychology may foster more efficient cooperation and competition among enterprises, enabling them to realise the value of co-creation and innovation breakthroughs.

## Introduction

Since Joseph Schumpeter referred to the concept of innovation in the early 20th century, an increasing number of research paradigms have been developed, and a paradigm shift has occurred from linear innovation models to innovation systems to innovation ecosystems. Linear innovation model considers innovation as a one-way process of successive development, as represented by the neoclassical school and endogenous growth theory (Li et al., 2014). The theory of innovation systems maintains the national innovation system as a central component and refers to influencing national innovation performance by changing state institutions at the macro level or by the interaction among actors at the micro level (Zeng et al., 2013). Technical corporations and interactions between enterprises and scientific research institutes (e.g., colleges and universities) are the central components of the national innovation system; innovation models are developing towards nonlinearity and symbiosis. With increasing prosperity of the social economy and the continuous innovation development of Silicon Valley, the original innovation system theory is no longer applicable; hence, innovation ecosystem theory has been proposed (Pique et al., 2018). The primary characteristic of innovation ecosystems theory is that innovation elements are freely combined and multiple symbioses exist. This engenders energy flow and material circulation with the external environment, thus forming an innovation ecosystem with self-organisational and openly symbiotic elements. Compared with the first two innovation paradigms, the innovation ecosystem approach pays more attention to the pluralistic symbiosis and value co-creation among innovation elements. Only by paying attention to the mechanisms of cooperation and competition among factors can knowledge and resources in the innovation ecosystem be absorbed, transformed and moved more efficiently, thus producing efficient innovation organisations and improving the economic performance of enterprises. Therefore, cooperation and competition among various elements in the innovation ecosystem are the core factor in realising value co-creation and innovation breakthroughs (Jones et al., 2021). Cooperation and competition have always been accompanied by the evolution and development of human beings, which is the key theme of evolutionary psychology research. Early in evolution, human beings formed cooperative alliances to hunt and defend against enemies, and at the same time challenged other cooperative alliances to compete for territory and food. Therefore, exploring the mechanisms underlying cooperation and competition in the innovation ecosystem from the perspective of evolutionary psychology would be conducive to promoting more efficient cooperation and competition among enterprises and thereby achieving innovation breakthroughs.

## A Review of Research on Innovation Ecosystems

Since the concept of the innovation ecosystem was proposed, it has not received a clear definition due to its vagueness, extensible nature and diversity of the associated research. However, as research has progressed, it has become clear that it is necessary to clearly define the concept of an innovation ecosystem in order to conduct theoretical discussions. Researchers have indicated that the innovation ecosystem represents an iterative update of the business ecosystem, which emphasises the acquisition of value, while the innovation ecosystem pays more attention to the creation of value (de Vasconcelos Gomes et al., 2018). The flow of knowledge and energy among actors in the innovation ecosystem will accelerate technological development and generate innovation. Some researchers have defined an innovation ecosystem as an organisational system with certain aspects of stability and independence, which is mutually dependent, symbiotic and includes the potential for mutual benefits among multiple innovation subjects, given common innovation elements, such as technology, talent and markets (Wu, 2014). These elements operate in a modular manner, rather than *via* hierarchical management, using collective investment to tie different actors within innovation (Jacobides et al., 2018). The relationship between innovation subjects is interdependent and complementary (Adner and Kapoor, 2010), while cooperation and competition among the actors in the innovation ecosystem guarantees value co-creation. The mechanisms through which the innovation ecosystem evolves are a topic of particular interest from the perspective of value creation (Wang and Zhu, 2018). Evolution of the innovation ecosystem is affected by the value-creating excess returns ratio, the cost of coordination, select deceptive and cooperation strategy benefits and costs, among other factors. From the perspective of purpose, governments and enterprises, as the core players in the innovation ecosystem, need to set goals to adapt the system to the environment, so as to smooth evolution (Gao and Tan, 2021). It has been suggested that a high level of scientific research in universities reduces the diversity of the innovation ecosystem (Arora et al., 2019). However, due to the diversity of research levels, there exist differences in the evolutionary mechanisms of innovation ecosystems. Regarding the spatial dimension, the research level of innovation ecosystems can be divided into industrial innovation ecosystems (Gawer and Cusumano, 2014), regional innovation ecosystems (Radziwon et al., 2017) and national innovation ecosystems (Fukuda and Watanabe, 2008). Some researchers have divided the innovation ecosystem into three levels: macro, mid and micro (Zhao and Zeng, 2014). At the macro level, the focus is on innovation subjects and their cooperation at the national level. The mid-level focuses on the industrial or regional innovation ecosystem, while the micro level primarily addresses activities at the organisation level, generally by analysing the individual behaviours of enterprises in the system. In addition to the conceptual study of the innovation ecosystem and the internal mechanisms through which it evolves, research has also considered the impact of external development on the evolution of the innovation ecosystem, including the role of public support (Radicic et al., 2020) and the social technology environment (Walrave et al., 2018). Some researchers have analysed the generation and evolution of the innovation ecosystem by disassembling the roles and functions of its different components (Dedehayir et al., 2018; Su et al., 2018). However, few studies have discussed the relationship between cooperation and competition within the innovation ecosystem nor the mechanisms underlying this relationship. Indeed, cooperation and competition between core enterprises and their complementary components are the core factors that determine whether an innovation ecosystem can be formed. Without cooperation, an innovation ecosystem cannot be formed. Therefore, it is necessary to explore cooperation and competition in an innovation ecosystem.

## Cooperation and Competition in the Innovation Ecosystem

The current rapid economic development and acceleration of the globalisation of multinational enterprises render it difficult for existing enterprises in isolation to obtain all necessary elements for innovation (Mei et al., 2014). Many companies are trying to establish or join innovation ecosystems to improve their innovation ability and market response. Large companies, such as Google and Apple, have established complex and increasingly effective innovation ecosystem based on their strong technical support and resource advantages (Moore, 1993). However, small businesses are not yet in a position to create an innovation ecosystem with themselves at its core; rather, they tend to participate in existing innovation ecosystems. However, whether a company is large or small, the success of innovation depends on close cooperation with partners (Su et al., 2018). In an innovation ecosystem, cooperation among innovation agents is the basis for ensuring smooth operation of the system. Cooperation is the main mechanism for creating value in the innovation ecosystem (Jones et al., 2021); its purpose is to enable agents to pursue common interests (Hannah and Eisenhardt, 2018). A major feature of the innovation ecosystem is complementarity among innovation subjects. Different subjects have different capabilities and economies and correspondingly show different innovation capabilities (Casadesus-Masanell and Yoffie, 2007). Cooperation can also solve the bottleneck problem in the innovation ecosystem (Ethiraj, 2007), which occurs when overall growth of the innovation ecosystem is restricted due to poor quality, poor performance or scarcity of some components (Baldwin, 2015). Such bottlenecks render other components and the whole system unable to realise their potential (Adner and Kapoor, 2016). Therefore, some bottleneck problems can be solved through cooperative research and development among different innovation entities. The evolutionary economics school posits that competition is the factor that drives innovation, and a competitive market environment gives birth to technological change and innovation, thus promoting economic growth (Winter and Nelson, 1982). Similarly, in an innovation ecosystem, competition is also a way to obtain value, and competition within the innovation ecosystem represents the enterprise’s pursuit of its own interests at the expense of others (Hannah and Eisenhardt, 2018). This type of competition generally occurs between companies that provide complementary goods or services. Enterprises obtain maximum value by limiting competition with respect to their goods or services while promoting competition regarding complementary outputs. The prerequisite to gain value through competition is to be the first to have market power (Santos and Eisenhardt, 2009). Enterprises can gain market power by entering the market early and then blocking the entry of other enterprises (Jacobides et al., 2006). For example, Intel and Microsoft were early microprocessor and operating system entries to the PC ecosystem, and restricted the entry of other enterprises by establishing component standards and utilising economies of scale (Gawer and Cusumano, 2002). Companies can also gain market power by reducing their dependence on companies that supply complementary goods or services. For example, the American music company MCA has benefited greatly from the talent industry by encouraging “fierce competition” among companies that provide such goods or services (Ferraro and Gurses, 2009). In the innovation ecosystem, innovation entities achieve mutual benefit by co-creating value through cooperation, but at the same time, they face the tension between competition and value ownership (Ansari et al., 2016; Ranganathan et al., 2018). Balancing the relationship between competition and cooperation is key to the stability and sustainability of an innovation ecosystem. However, competition and cooperation exist not only between innovation agents but also in different ways at multiple ecosystem levels, with certain complexities (Hannah and Eisenhardt, 2018). Companies often compete for market share, but also cooperate to defend, develop and expand their ecosystems. However, if companies cooperate too much, they may not be sufficiently profitable to survive. In contrast, if competition among enterprises is too fierce, an innovation ecosystem may not be formed (Ozcan and Santos, 2015). Therefore, in an innovation ecosystem, the relationship between cooperation and competition presents a dynamic balance. Researchers have found that competitive innovation agents and companies tend to continue to cooperate: the greater the expected level of benefit for the innovation agent, the greater the possibility of the firm maintaining cooperation in the presence of competitive conflicts (Jones et al., 2021). The innovation ecosystem is a dynamic and complex innovation network composed of complementary core enterprises and upstream and downstream suppliers. Competition and cooperation within the system are complex and variable and cannot be explained by linear thinking. This paper is based on the perspective of evolutionary psychology, therefore only explores cooperation and competition based on the principles of value co-creation and reciprocal altruism, so that the relationship between cooperation and competition in the innovation ecosystem can be explored from a psychological perspective, opening up research ideas.

## The Role of Evolutionary Psychology

Since the birth of human society, cooperation has been accompanied by human evolution and development. Early in evolution, human beings were forced to cooperate in order to survive the harsh natural environment, and thus survive and reproduce (Cohen and Bernard, 2013). According to evolutionary psychology, cooperative behaviour is motivated by reciprocal altruism.

As different from altruism, in which altruists sacrifice their own interests to help others, in reciprocal altruism, the altruist receives a reward from the recipient at some point in the future, so that both parties benefit. In the field of economics, reciprocal altruism is often termed “win-win”, that is the reward for cooperation is higher than its cost to the two parties. Cooperation under the theory of reciprocal altruism is not unconditional. Trust is an important factor that determines whether cooperation can be achieved. In reciprocal cooperation, cheating can readily occur; that is, some people obtain but do not provide benefits (Cosmides and Tooby, 1992, 2005). Such “cheating behaviour” threatens the evolution of cooperation. To address the “cheating threat”, individuals require psychological mechanisms that enable them to detect the likelihood of cheating before choosing partners and thereby avoid being subject to cheating. In addition to cooperation between individuals, a cooperative alliance is also a form of cooperative behaviour, which refers to the collective action taken by more than two individuals to achieve a specific goal. In primitive societies, humans formed cooperative alliances to hunt, fend off attacks and build houses. Like cooperative behaviour, cooperative alliances face the threat of cheating and free-riding; only by addressing these two issues can such alliances continue to evolve. According to evolutionary psychology, individual competitiveness is a universal psychological mechanism developed during human evolution. Early in evolution, humans faced many threats and needed to adopt particular psychological mechanisms to avoid being harmed by other groups or individuals. Individuals in competitive situations experience increased adrenaline and focus; thus, the psychological mechanisms underlying competition were formed. Through competition, individuals can occupy resources, improve social status and power, and more importantly, successful individuals can gain dominance. An individual who has attained a dominant level has improved access to critical resources versus other individuals; the greater the dominance of an individual, the more critical resources they have attained and vice versa (Cummins, 1998). Competition does not rely on force and threats. One can also win competition by using one’s own network of relations and knowledge and skills, thus winning “prestige”. In evolutionary psychology, “prestige” refers to the “willing obedience” of others. A person with prestige is sought by others for professional guidance (Henrich and Gil-White, 2001) or is a person from whom others can obtain certain reproductive benefits. Persons in lower social classes often approach or imitate persons with prestige. In modern society, to build prestige and win competitions, it is necessary to exhibit extraordinary ability, while being willing to sacrifice personal for overall interests (Anderson and Kilduff, 2009). As distinct from previous studies, this study adopts the perspectives and theories of reciprocal altruism, trust and free-riding from evolutionary psychology to explain the mechanisms that influence cooperation and competition in an innovation ecosystem. Based on the principle of reciprocal altruism, core enterprises in the innovation ecosystem connect with complementary enterprises for mutual learning, and to utilise the ability and resource advantages of each agent, to foster common interests and achieve a win-win result. In evolutionary psychology, cheating and free-riding are important factors in the dissociation of cooperative alliances. Similarly, in an innovation ecosystem, the success of innovation depends on mutual trust between innovative agents (Chesbrough et al., 2014). Astling emphasises that supporting innovation should follow the core values of five elements: ask, take risks, be open, be patient and trust. Therefore, trust plays a fundamental role in an innovation ecosystem. Free-riding behaviour of actors in the innovation ecosystem can create a bottleneck threat to the entire system. As mentioned above, actors with poor quality, poor performance or scarcity in an innovation ecosystem can affect other actors or fail to realise their potential in the entire system. Accordingly, the identification and elimination of free-rider companies are necessary to ensure smooth operation of the innovation ecosystem.

In groups, in addition to cooperation, another important tool commonly used for most strategic matters is competition. Especially in task-oriented teams, competition can stimulate employees to improve efficiency, enhance team achievement and maintain organisational competitiveness and market share (Tjosvold, 1998). In evolutionary psychology, individuals gain prestige through competition to attract more individuals, while an innovation ecosystem is an interest chain formed around the core enterprise by many different complementary enterprises; small enterprises join the ecosystem for value co-creation because of the prestige of the core enterprise. It has been pointed out that intra-group competition can undermine in-group preferences, and the way to solve this problem is to focus more on individual relationships among group members rather than strengthening the ties between individuals and the collective (Zuo et al., 2019). Therefore, this warns that while core firms compete to acquire core technologies, large networks of relationships, and preferred resources, and work together to achieve innovation with the assistance of complementary firms, it is important to balance intra-system cooperation and competition, sacrifice “individual interests” appropriately and maintain the ties of intra-team members in order to maintain the innovation ecosystem’s. The balance between cooperation and competition within the system, the appropriate sacrifice of “personal gain”, and the maintenance of linkages among team members is necessary to maintain the stability and sustainability of the innovation ecosystem.

## Research and Prospects

This study explores the relationship between cooperation and competition in an innovation ecosystem from the perspective of evolutionary psychology. Cooperation can not only lead to achieving common goals in the system, but also solve the bottleneck problem. Similarly, competition can continuously improve the competitiveness of the innovation ecosystem and enable members to obtain greater market share and resources. This paper also analyses the mechanisms by which cooperation and competition form the perspective of evolutionary psychology. It is found that trust and opposition to free-riding are not only the main factors that prevent the collapse of cooperative alliances but are also important factors in maintaining the development of an innovation ecosystem. Second, the purpose of competition is to acquire resources and to win social status and prestige. Cooperation between and within the system can create better conditions for competition, and competition within the system will not destroy cooperation. On the contrary, cooperation can continue, provided that the core enterprise has high profit expectations associated with cooperation. Using evolutionary psychology of “reciprocal altruism” and “trust” “free-rider” point of view and theory explains the innovation ecosystem of cooperation and competition, and also explains the evolutionary psychology of all attainments in psychology, not only in the field of other disciplines will but also be able to play the role of theoretical support, gradually formed from theory to practice verify the breakthrough. This study is based on the innovation ecosystem in each main body between cooperation and competition, with no distinction between components upstream and downstream components in detail, and does not have to explore different levels of cooperation and competition; the innovation ecosystem complexity of an innovation ecosystem will result in certain differences related to investigation, and future studies will be discussed in more detail.

## Author Contributions

The author confirms being the sole contributor of this work and has approved it for publication.

## Conflict of Interest

The author declares that the research was conducted in the absence of any commercial or financial relationships that could be construed as a potential conflict of interest.

## Publisher’s Note

All claims expressed in this article are solely those of the authors and do not necessarily represent those of their affiliated organizations, or those of the publisher, the editors and the reviewers. Any product that may be evaluated in this article, or claim that may be made by its manufacturer, is not guaranteed or endorsed by the publisher.
